# Doctors in Canada

**Published:** 1882-12-20

**Authors:** 


DOCTORS IN CANADA.—It is said that the proportion of Doctors to the population in Canada is as one to 1,200, while in the United States they are as one to 600.

